# PD13 - Gender differences in rhinitic children

**DOI:** 10.1186/2045-7022-4-S1-P13

**Published:** 2014-02-28

**Authors:** Giuliana Ferrante, Velia Malizia, Maria Tornatore, Laura Montalbano, Roberta Antona, Giovanni Corsello, Stefania La Grutta

**Affiliations:** 1 Department of Science for Mother and Child Health Promotion, University of Palermo, Palermo, Italy; 2 Institute of Biomedicine and Molecular Immunology IBIM, National Research Council, Palermo, Italy

## 

Gender differential effects on rhinitis are infrequently studied.

**Aim** of our study is to assess gender differences in host and environmental characteristics and in rhinitis severity level within the IBIM Pulmonary and Allergy Pediatric Clinic. A series of rhinitic (R) patients (September 2011 - May 2013) were investigated through standardized questionnaire and spirometry. Statistical analyses were performed with SPSS.

**Preliminary results** refer to 122 R patients: 77 males (M) (63.1%) and 45 females (F) (36.9%); age (years): 9.23 ± 3.42M *vs* 9.38 ± 3.02F; maternal history of rhinitis: 45.5%M *vs* 32.3%F (p<0.090); exposure to maternal smoking during pregnancy:15.6%M *vs* 2.2%F (p<0.021); exposure to passive smoke: 49.4%M *vs* 33.3%F (p<0.086); exposure to only current maternal smoke: 24.7%M *vs* 11.1%F (p<0.070); current exposure to pet: 31.2%M *vs* 15.6%F (p<0.057); exclusive breast feeding (4mos): 33.8%M *vs* 53.3%F (p<0.034); BMI (Kg/m^2^):18.98±3.99M *vs* 17.95±2.94F (p<0.133); being overweight: 39%M *vs* 24.4%F (p<0.083). After stratifying by presence/absence of asthma, in those with R only (57, 46.7%): 42%M vs 53.3%F (p<0.267);VAS (mean±s.d.): 8.18±1.46 M *vs* 7.60±1.71F (p<0.099); PSQI (mean±s.d.): 2.33±1.53M *vs* 1.44±0.73F (p<0.009); FVC (%Pred) (mean±s.d.): 98.14±10.51M *vs* 103.27±7.83F (p<0.068); in those with rhinitis and asthma (RA, 65. 53.3%): 57.1%M *vs* 46.7%F (p<0.267); asthma severity level: intermittent, 32.5%M *vs* 11.1%F (p<0.008); moderate persistent, 9.1%M *vs* 15.6%F (p<0.063); rhinitis severity level: mild persistent 33.8% RA *vs* 17.5% R-only (p<0.041);VAS (mean±s.d.): 6.91±1.57 M *vs* 8.50±1.68F (p<0.010); food allergy 36.4%M *vs* 4.8%F (p<0.008).

**In conclusion**, we have shown in a consecutive series of rhinitic patients that male gender is mainly associated with more frequent exposure to environmental and parental risk factor, burden of disease, pulmonary function tests and co-morbididy, but also with less severe rhinitis level. Further analyses on a larger series of pediatric patients are needed in order to assess the impact of gender differences on rhinitis management.

